# Prevalence and Clinical Course in Invasive Infections with Meningococcal Endotoxin Variants

**DOI:** 10.1371/journal.pone.0049295

**Published:** 2012-11-29

**Authors:** Gerwin D. Rodenburg, Floris Fransen, Debby Bogaert, Kim Schipper, Rolf H. H. Groenwold, Hendrik Jan Hamstra, Brenda M. Westerhuis, Diederik van de Beek, Peter van der Ley, Elisabeth A. M. Sanders, Arie van der Ende

**Affiliations:** 1 Department of Pediatric Immunology and Infectious Diseases, Wilhelmina Children's Hospital, University Medical Center Utrecht, The Netherlands; 2 Unit Vaccinology, National Institute for Public Health and the Environment, Bilthoven, The Netherlands; 3 Department of Immunology and Infectious Diseases, Utrecht University, Utrecht, The Netherlands; 4 Department of Medical Microbiology, Academic Medical Center Amsterdam, Center for Infection and Immunity Amsterdam, Amsterdam, The Netherlands; 5 Julius Center for Health Sciences and Primary Care, University Medical Center Utrecht, Utrecht, The Netherlands; 6 Department of Neurology, Center for Infection and Immunity Amsterdam (CINIMA), Academic Medical Center, Amsterdam, The Netherlands; 7 Netherlands Reference Laboratory for Bacterial Meningitis, Academic Medical Center, Amsterdam, The Netherlands; Universidad Nacional de La Plata., Argentina

## Abstract

**Background:**

Meningococci produce a penta-acylated instead of hexa-acylated lipid A when their *lpxL1* gene is inactivated. Meningococcal strains with such lipid A endotoxin variants have been found previously in adult meningitis patients, where they caused less blood coagulopathy because of decreased TLR4 activation.

**Methods:**

A cohort of 448 isolates from patients with invasive meningococcal disease in the Netherlands were screened for the ability to induce IL-6 in monocytic cell Mono Mac 6 cells. The *lpxL1* gene was sequenced of isolates, which show poor capacity to induce IL-6.. Clinical characteristics of patients were retrieved from hospital records.

**Results:**

Of 448 patients, 29 (6.5%) were infected with meningococci expressing a lipid A variant strain. Lipid A variation was not associated with a specific serogroup or genotype. Infections with lipid A variants were associated with older age (19.3 vs. 5.9 (median) years, p = 0.007) and higher prevalence of underlying comorbidities (39% vs. 17%; p = 0.004) compared to wild-type strains. Patients infected with lipid A variant strains had less severe infections like meningitis or shock (OR 0.23; 95%CI 0.09–0.58) and were less often admitted to intensive care (OR 0.21; 95%CI 0.07–0.60) compared to wild-type strains, independent of age, underlying comorbidities or strain characteristics.

**Conclusions:**

In adults with meningococcal disease lipid A variation is rather common. Infection with penta-acylated lipid A variant meningococci is associated with a less severe disease course.

## Introduction

*Neisseria meningitidis* is an obligate commensal of the human nasopharyngeal mucosa and is found in 10–20% of healthy individuals [1], [2]. Meningococcal colonization usually is observed as asymptomatic carrier state, but may progress towards invasive bacterial infections varying from transient blood infection (meningococcaemia), to meningitis and fulminant septic shock with high mortality and morbidity rates [3], [4]. Incidence of meningococcal disease shows its primary peak in children aged <5 years of age with up to 20 cases per 100,000 per year in infants under 1 year of age [5]. This increased susceptibility has been attributed to an immature immune system and lack of protective antibodies [6], [7]. A second peak in meningococcal disease incidence is in adolescents and young adults probably due to behavior and environmental conditions [8], [9]. Overall, capsule serogroups that cause most invasive disease (A, B, C, W-135, X and Y) vary greatly between carrier and disease isolates [5], [10]. Besides the polysaccharide capsule, other meningococcal genetic determinants may be decisive for invasiveness, as hyper-invasive lineages causing disease are less frequently found among carrier isolates [1], [2].

The major virulence factor in meningococcal disease is endotoxin consisting of lipopolysaccharide (LPS), a potent activator of the innate immune system via Toll-like receptor 4 (TLR4) in combination with co-receptors MD-2 and CD14 [11], [12]. The TLR4 receptor complex mainly recognizes the lipid A moiety of the LPS molecule, and the position, length and number of fatty acyl chains in the lipid A determine the degree of TLR4 activation [13]. Broad stimulation of TLR4 in patients with invasive meningococcal disease leads to acute systemic inflammation followed by hypotension, circulatory collapse and shock. Upregulation of tissue factor also leads to activation of the coagulation system and disseminated intravascular coagulation [3], [14]. Recently, we reported that a surprisingly large fraction of meningococcal clinical isolates from adult meningitis patients, had endotoxin with underacylated lipid A due to mutations in the *lpxL1* gene [15]. The *lpxL1* gene of these isolates mostly had a phase-variable *lpxL1* mutation, suggesting that under certain conditions lipid A variation is beneficial to the meningococcus. In vitro, the variant strains showed decreased TLR4-mediated induction of cytokines IL-6, TNF-α and IL-1β in comparison to hexa-acylated wild-type strains [15], [16]. The resulting low-activity lipid A may therefore have a role in virulence by helping the bacteria to evade the innate immune system. We also showed that in adults with meningococcal meningitis, infection with lipid A variants is associated with a milder clinical course of disease with reduced coagulopathy and systemic inflammation [15]. However, impact and prevalence of lipid A variants in other clinical disease syndromes and age categories, including the youngest, is currently unknown, as well as the prevalence among carriage strains.

In the current study, we explored the frequencies of lipid A variants in a large cohort of meningococcal disease isolates collected over recent years. We compared the distribution of lipid A variants among meningococcal serogroups and genotypes and investigated clinical impact of lipid A variants in various disease syndromes and at all ages.

## Methods

### Ethics

The study was approved by the medical ethics committee of the University Medical Center Utrecht (Medisch Ethische toetsingscommissie UMC Utrecht; www.umcutrecht.nl/subsite/Medisch +Ethische+Toetsingscommissie). The medical ethics committee concluded at 08-11-2007 the research-project did not fall within the ambit of the WMO (Medical Research Involving Human Subjects Act) because it is part of the national surveillance program. The committee concluded informed consent would cause substantial bias and missing data that would hamper good estimates on national burden of disease. The project was carried out by the code ‘goed gedrag’ and ‘goed gebruik’ (www.fmwv.nl).

### Meningococcal isolates

The Netherlands Reference Laboratory for Bacterial Meningitis (NRLBM) has a laboratory-based nationwide surveillance system that collects bacterial isolates from blood, cerebrospinal fluid (CSF) and other normally sterile bodily fluids. All cases with invasive meningococcal disease reported by nine sentinel laboratories between June 1^th^ 2001 and May 31^th^ 2006 were included for analyses in this retrospective cohort study. Serogrouping and MLST was performed as described elsewhere [17], clonal complexes were designated according to the online MLST-database [18].

### Lipid A variant detection

To identify meningococcal isolates as lipid A variants, isolates were tested for their capacity to induce the pro-inflammatory cytokine IL-6 [15] by investigators blinded for patients data. In summary, heat-inactivated bacterial suspensions were added to the human monocytic cell line Mono Mac 6 [19]. After incubation the amount of IL-6 in the supernatant was determined by ELISA. We have previously shown that when the bacterial suspension is diluted to an OD620 of 0.001, *lpxL1* mutant bacteria can be readily distinguished by their strongly reduced level of IL-6 induction [15].

### Patient characteristics

Clinical information of invasive disease infections was retrospectively extracted from hospital records using an anonymous standard data collection form and was performed in accordance with the Dutch privacy legislation by investigators blinded for the results of the lipid A variant assessment. If retrievable from medical record, clinical syndromes were classified in four categories: shock, shock with meningitis, meningitis without shock or no shock or meningitis. Meningitis was defined as a clinical diagnosis of meningitis in combination with a blood or CSF culture positive for *N. meningitidis*. Shock was defined as a clinical diagnosis of shock by the treating physician at admittance or during hospitalization. Information about the clinical presentation of the patient on admission was collected together with laboratory parameters from blood taken within the first 12 hours after hospital admission and as well as data on the course of disease. As underlying conditions were included immunocompromising conditions like primary immunodeficiency, HIV, lymphoma, leukemia, stem cell transplantation, current immunosuppressive therapy for malignancy or autoimmune disease, asplenia, sickle cell disease, renal insufficiency/need for dialysis, and nephritic syndrome. Other co-morbidities included were current malignancies, chronic obstructive pulmonary disease/asthma (among persons aged 5 yrs and older), diabetes mellitus, cardiovascular disease (myocardial infarction, coronary artery condition, cerebrovascular accident/transient ischemic attack, cardiomyopathy/heart failure, heart valve disease, presence of cerebral/abdominal/thoracic aneurysms), thyroid disease, liver disease, intravenous drug use and alcohol abuse. For cases in children, also premature birth (<37 weeks for 0–1-yr-olds and <32 for 0–4-yr-olds), serious perinatal complications for 0–1-yr-olds, congenital conditions/syndromes were recorded. Case-fatality was defined as in-hospital death or death within 30 days after collection of the first blood/CSF culture positive for *N. meningitidis*.

### Statistical analysis

Proportions were tested using Chi-square test or Fisher's exact tests as appropriate. Continuous outcomes were tested usingMann Whitney U test All statistical tests were two-tailed, p-values of less than 0.05 were considered significant. For the clinical characteristics, for each significantly different dichotomous outcome a multivariable logistic regression model was fitted to estimate the association between determinants and the outcome. Lipid A variation, gender, underlying comorbidities, serogroup (B; C; others) and clonal complexes (cc11; cc32; cc41/44; others) were included as categorical determinants. Age was included as continuous determinant. Statistical analyses were performed with SPSS 15.0, figures were made with Excel 2007.

## Results

### Lipid A variants among meningococcal isolates from patients

In total 467 cases of invasive meningococcal disease were reported to NRLBM, and of 448 cases (96%) both clinical data and in vitro IL-6 induction could be studied. Twenty-nine isolates (6.5%) had a decreased potential to induce IL-6, which was shown previously to distinguish between lipid A variant and wild-type strains. Of 467 isolates, 20 (4%) were used in the previous study (15), 19 with wild-type lipid A and 1 with a lipid A variant. Medical records were found for 18 of these 20 strains. The two isolates for which no medical data could be retrieved were both wild-type strains.

The proportion of serogroup B isolates among lipid A variant isolates was lower than that among wild-type isolates (62% vs. 79%, respectively; *p* = 0.03), while the proportion of serogroup C isolates was higher among lipid A variants than among the wild-type isolates (38% vs. 20%, respectively; *p* = 0.02) (Table 1). The proportion of clonal complex cc11 among isolates with a lipid A variant was higher than among wild-type isolates (35% vs. 16%, respectively; *p* = 0.01) (Table 2). Three isolates with a lipid A variant were of cc 451, while this clonal complex was not observed among wild-type isolates (*p* = 0.0003).

**Table 1:** Serogroup distribution of lipid A variants among isolates from patients with invasive meningococcal disease.

Serogroup	Wild type	Lipid A variant	*p* value^a^
	N = 419	N = 29	
B	331 (79%)	18 (62%)	**0.03**
C	84 (20%)	11 (38%)	**0.02**
29^E^ and W135	4 (1%)	-	

a Proportions were tested with Chi-square test or Fisher's exact tests, as appropriate. All p values are 2 sided; Significant correlations (p<0.05) are depicted in bold.

**Table 2:** Clonal complex distribution of lipid A variants among isolates from patients with invasive meningococcal disease.

Clonalcomplex	Wild type	Lipid A variant	*p* value^a^
	N = 409	N = 29	
cc41/44	206 (50%)	11 (38%)	0.19
cc32	69 (16%)	2 (7%)	0.20
cc11	66 (16%)	10 (34%)	**0.01**
cc213	13 (3%)	2 (7%)	0.26
cc35	2 (0.5%)	1 (3%)	0**.19**
cc461	-	3 (10.3%)	**0.0003**
Others	63# (15%)	-	

a Proportions were tested with Chi-square test or Fisher's exact tests, as appropriate, all p values are 2 sided; Significant correlations (p<0.05) are depicted in bold.

# Comprising 12 clonal complexes.

### LpxL1 mutations

All low IL-6 inducing strains found in disease isolates revealed *lpxL1* mutations in sequence analyses (See supporting information Figure S1 for type and positions of mutations). Nine different *lpxL1* mutations were found, with 19/29 (66%) being reversible, phase-variable mutations (mutation type III, IV or V).

### Patient characteristics

**Figure 1:**
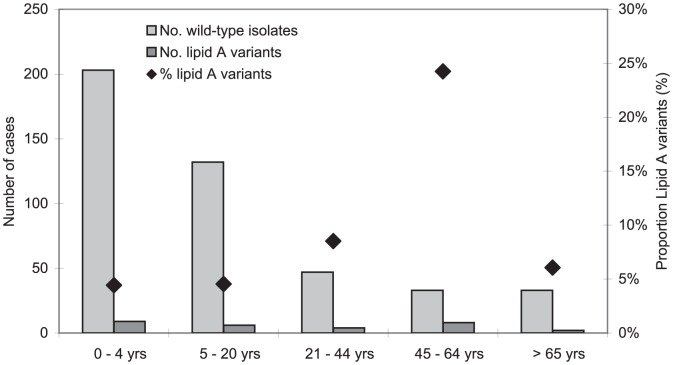
Number of wild-type or lipid A variant disease cases in different age-categories (bars) and proportion of lipid A strains (dots).

Of the 448 patients with invasive meningococcal disease of whom data from the medical records were available, the median age was 5.9 years (interquartile range (IQR) 1.8–18.9) in case of wild-type meningococcal infection vs. 19.3 years (IQR 4.6–50.5) for the lipid A variant infected patients (p = 0.007). The proportion of lipid A variant strains in invasive disease was lowest in children and adolescents under 20 years of age i.e. 15/335 (4%) and rose gradually to 24% (8/33 cases) among patients 45–64 years of age (Figure 1). All patients required hospitalization on admittance except for 2 patients, who were presented at the acute medical ward but were sent home because of mild symptoms. Both patients were found to have been infected with lipid A variant strains.

### Clinical characteristics

Of 29 patients infected with lipid A variants, 11 (39%), had underlying comorbidities versus 71/406 (17%) patients infected with wild-type meningococci (p = 0.004) (Table 3; see supporting information Table S1 for disease characteristics of each lipid A infected patient). A milder clinical presentation on admission was seen after infection with a lipid A variant; less patients presented with fever (14/25 [56%] vs. 253/330 [77%]; p = 0.02) and petechiae (14/28 [50%] vs. 271/397 [68%]; p = 0.047) compared to patients infected with wild-type strains (Table 1). The proportion of patients with a duration of symptoms less than one day before admittance was lower among patients infected with lipid A variants than among patients infected with wild-type meningococci (1/23 [4%] vs. 88/378 [23%], respectively; p = 0.04).

**Table 3:** Clinical manifestation on admittance and disease course in patients with meningococcal disease due to wild type or lipid A variant strains.

	Wild type	Lipid A variant	p-value^a^
Patient characteristics	N = 419	N = 29	
Age at presentation, years (IQR)	5.9 (1.8–18.9)	19.3 (4.6–50.5)	**0.007**
Male, *n (%)*	218/419 (52%)	12/29 (41%)	0.27
Underlying comorbidity, *n (%)*	71/406 (17%)	11/28 (39%)	**0.004**
**Clinical manifestation at admission**			
Disease syndrome: Meningitis, *n (%)*	364/406 (90%)	17/28 (61%)	**0.0001**
Symptoms before admittance <1 day, *n (%)*	88/378 (23%)	1/23 (4%)	**0.04**
Symptoms before admittance ≥1 week, *n (%)*	24/369 (7%)	3/24 (14%)	0.22
Fever, temperature >38°C, *n (%)*	253/330 (77%)	14/25 (56%)	**0.02**
Petechiae, *n (%)*	271/397 (68%)	14/28 (50%)	**0.047**
**Clinical Course during admission**			
DIS/MOF, *n (%)*	43/388 (11%)	1/28 (4%)	0.34
Active ventilation, *n (%)*	86/386 (22%)	1/28 (4%)	**0.02**
Days in hospital, median (IQR)	9 (8–12)	9 (7–16)	0.80
ICU admittance, *n (%)*	150/393 (38%)	5/29 (17%)	**0.03**
Mortality, *n (%)*	33/390 (8%)	3/29 (10%)	0.73

DIS/MOF, Multiple organ failure or disseminated intravascular coagulation; IQR, Interquartile range.

a Proportions were tested with Chi-square test or Fisher's exact tests, as appropriate, all p values are 2 sided. Continuous outcomes were tested with nonparametric tests; Significant correlations (p<0.05) are depicted in bold.

In laboratory investigations, only differences in prothrombin time (medians, 13 (IQR 2–17) vs. 18 (IQR 14–23); p = 0.01) and in levels of serum lactate (medians, 2.0 (1.1–2.3) vs. 3.4 (IQR 2.2–5.9); p = 0.03) were observed between lipid A variant infections and infections with wild-type strains (Table 4). During the course of infection, patients with infection due to lipid A variants were less likely to be admitted to the ICU (5/29 [17%] vs. 150/393 [38%]; *p* = 0.03) and to require mechanical ventilation (1/28 [4%] vs. 86/386 [22%]; *p* = 0.02) but there was no difference in mortality.

**Table 4:** Blood investigations within 12 hours after admittance in patients with meningococcal disease due to wild-type of lipid A variant strains.

	Wild-type		Lipid A variant		p-value^a^
Parameters	Median (IQR)	N	Median (IQR)	N	
**Infectious**					
White cell count, 10^9^/L (lQR)	15.7 (9.1–23.7)	388	16.4 (13.6–23.1)	28	0.30
Neutrophil count, 10^9^/L (lQR)	12.4 (6.2–20.4)	254	12.8 (8.9–22.4)	17	0.43
-Immature	1.7 (0.58–3.8)	223	1.8 (0.1–2.9)	11	0.50
-Mature	11.0 (4.9–17.2)	261	11.9 (8.3–16.2)	16	0.61
Lymphocyte count, 10^9^/L (lQR)	1.5 (0.9–2.3)	290	1.3 (0.7–2.1)	17	0.40
CRP, mg/dl (lQR)	149 (81–229)	375	126 (75–230)	26	0.48
**Coagulation**					
Thrombocyte count, 10^9^/L (lQR)	216 (163–277)	372	237 (161–282)	25	0.59
PT, sec (IQR)	18 (14–23)	111	13 (2–17)	9	**0.01**
aPTT, sec (IQR)	40 (33–52)	145	40 (35–57)	8	0.81
**Other parameters**					
Hemoglobine, mmol/l (IQR)	7.5 (6.9–8.3)	383	8.0 (7.3–8.4)	28	0.09
Sodium, mmol/l (IQR)	136 (134–138)	358	136 (133–138)	24	0.49
Potassium, mmol/l (IQR)	3.7 (3.3–4.0)	358	3.9 (3.5–4.2)	23	0.09
Glucose, mmol/l (IQR)	6.9 (5.7–8.3)	322	7.4 (5.7–9.8)	23	0.17
Lactate, µmol/l (IQR)	3.4 (2.2–5.9)	118	2.0 (1.1–2.3)	4	**0.03**
Creatinine, µmol/l (IQR)	65 (42–97)	327	69 (56–100)	24	0.41
Urea, mmol/l (IQR)	5.3 (4.2–7.3)	305	5.7 (4.1–6.3)	20	0.84

IQR, Interquartile range.

a P values were calculated by nonparametric tests; all p values are 2 sided.

Clinical manifestations that show a significant correlation (p<0.05) with lipid A variant meningococci are depicted in bold. PT means Prothrombin Time; aPTT is activated Partial Thromboplastin Time.

Multivariable analyses showed that lipid A variant infected patients developed less frequenlty meningitis or shock (OR 0.23; 95% CI 0.09–0.58; *p* = 0.002) (Table 5), independent of age, gender, underlying comorbidities, serogroup or clonal complex. Also they were less likely to be actively ventilated (OR 0.11; 95% CI 0.01–0.81; *p* = 0.03) or admitted to the ICU (OR 0.21; 95% CI 0.07–0.60; *p* = 0.004) For fever, petechiae and symptoms less then one day before admittance no impact of lipid A variety could be proven.

**Table 5:** Multivariable analyses for impact of infection with lipid A variants on different outcome variables.

Outcome variable	Adjusted OR (95% CI)^a^	p-value
Disease syndrome; Meningitis or Shock	**0.23 (0.09–0.58)**	**0.002**
Symptoms before admittance <1 day	0.16 (0.02–1.23)	0.08
Fever; temperature >38°C	0.47 (0.19–1.13)	0.09
Petechiae	0.61 (0.27–1.34)	0.23
Active ventilation	**0.11 (0.01–0.81)**	**0.03**
ICU admittance	**0.21 (0.07–0.60)**	**0.004**

a Odd ratio adjusted for age, gender, underlying comorbidities, serogroup and clonal complex.

## Discussion

LPS, also named endotoxin, is a very potent activator of the immune system present in whole bacteria or in outer membrane vesicles released by meningococci (3). Variation in lipid A and LPS-structure has been described for several bacteria and is thought to enable pathogens to better evade host immunity [20], [21]. Among patients with invasive meningococcal disease, we found frequencies of lipid A variants up to 24% depending on age. Meningococcal infection due to lipid A variants seems rather common among adults and vulnerable patients with underlying comorbidities and was associated with a less severe course of disease. Infections with lipid A variants do not seem associated with any particular meningococcal serogroup or genotype. We previously showed that the underacylated LPS strains resulted in decreased TLR4-mediated induction of cytokines by macrophages [15], [16]. We also reported a diversity in phenotypes of isolates obtained from an individual patient from different anatomical sites in 3/40 (8%) cases and *in vitro* phase-variation was observed to rarely occur, while *in vitro* phase-variation was observed to rarely occur [15]. This means the fraction of patients with mutant *lpxL1* isolates is potentially higher. The majority of the lipid A variant strains in the present study had reversible mutations. This suggests selective advantage of the meningococcus with penta-acylated lipid A during invasion of the host while meningococci with hexa-acylated lipid A are in favor during colonization of the nasopharynx in carriage. In accordance with this interpretation, we observed a very low frequency of lipid A variant strains among carrier isolates (A van der Ende, unpublished data). This way, meningococci seem more invasive for the vulnerable host with underlying comorbidities rendering him susceptible for invasive bacterial disease.

In contrast with wild-type invasive meningococcal disease, infections with lipid A variant strains did not show the typical age-dependent decrease in incidence-rate after puberty. The decrease in disease incidence is generally ascribed to increasing levels of adaptive immunity i.e. protective antibodies. In young children adaptive and innate immune components are not yet fully matured and innate responses to LPS in neonates have been described to be impaired as well [22], [23]. Also host mechanisms like complement and leukocyte activation can differ [24]. Possibly, next to protective antibodies, clearing of meningococci from the blood may also largely depend on TLR 4 activation. The decreased activation of TLR4 signaling by LPS variations might thus be a possible mechanism for enhanced survival of lipid A variants in the blood stream. This has been described for *Bortella parapertussis*: in animals passively immunized with antibodies, this bacterium survived and grew efficiently [25]. When TLR4 agonist was added, *B. parapertussis* was cleared. Secondly, TLR-4 activation may be vital for antibody function. TLR4 signalling for example proved to be essential for FcRIII signalling, through a connection at receptor level between TLR4 and FcRIII pathways [26]. Together, this indicates that vaccine failures may occur when vaccinated hosts are infected by meningococcal lipid A variants with penta-acylated lipid A due to reduced induction of adaptive immunity. The potential association between the development of specific immunity and lipid A variant frequency should be further investigated.

Lipid A variant and wild-type meningococcal infections cause a different disease course. Although causality between lipid A variance and clinical impact is difficult to prove, the observed differences in our study are in line with the assumption that the lipid A moiety is one of the main triggers of the host's systemic inflammatory reaction. We found that infection with lipid A variant strains is an independent determinant of a milder course of disease by several clinical characteristics. In the previously published, prospective study we found that in adults with meningococcal meningitis, infection with lipid A variants is associated with a milder clinical course of disease with reduced coagulopathy. However, in the current study we could not prove in multivariate analyses influence on amount of petechiae and coagulation in patients infected with lipid A variants which would support the notion that the lipid A variants have reduced potential for activation of tissue-factor mediated coagulopathy [27]. Possibly, differences in time of collection (at admission vs. within 24 hours after admission) or the design of the study (retrospective vs. prospective) may partly explain this difference. Bias by earlier admission of lipid A variants seems unlikely, since patients with lipid A variant infections tended to have symptoms for a longer period before admission, possibly due to a delayed host inflammatory response as is described for *B. parapertussis* [25]. Also a higher proportion of underlying comorbidities was found in patients with lipid A variant strains. Higher vulnerability can explain the mortality-rates in lipid A variant infected patients, because all three deceased patients with lipid A variants had underlying comorbidities.

Some important limitations of our study should be acknowledged. First, despite the large screening cohorts, numbers of lipid A variants are relatively small, leaving some uncertainties for comparative analyses. Especially in the youngest age-categories, small numbers result in limited precision. However, for the older age-categories differences after infection with lipid A variant strains or wild-type strains seem consistent for most parameters tested, also after correcting for other potential predictors. Second, clinical data were collected retrospectively. Therefore for some clinical parameters like shock we had sometimes to rely on physicians notes without objective parameters. Proportions of shock therefore might be overestimated compared with earlier published studies [4]. However, most clinical parameters and essential laboratory values could be retrieved and in only 2% of the cases no clinical information was available. Nevertheless, some laboratory parameters like lactate should be interpreted with caution since only for a limited number of cases data were available.

In conclusion, we show that a significant proportion of meningococcal disease is caused by meningococci with underacylated lipid A. Possibly, due to inefficient stimulation of TLR4, lipid A variant meningococci poorly stimulate the inflammatory response and are better able to avoid clearance. If so, patients infected with lipid A variants endure milder symptoms with less systemic inflammation. Lipid A variant infections are very common among adults and in particular vulnerable patients with underlying comorbidities. These findings emphasize further study of this naturally occurring endotoxin variant in invasive disease but also in carriage.

## Supporting Information

Figure S1 **Type and position of mutations in the** ***lpxL1*** **-gene of strain MC58.**
(EPS)

Table S1 **Strain and patient characteristics of meningococcal lipid A variant disease isolates.**
(DOC)
