# Supplementary material for: Prevalence and Clinical Course in Invasive Infections with Meningococcal Endotoxin Variants
Source: PLoS One. 2012 Nov 29;7(11):e49295. doi: 10.1371/journal.pone.0049295 (PMC3510230; doi:10.1371/journal.pone.0049295)
Supplement: Table S1 — Strain and patient characteristics of meningococcal lipid A variant disease isolates. (DOC) [file pone.0049295.s002.doc]

| **Strain no.** | **Age (yrs)** | **Disease syndrome** | **Comorbidities** | **Mortality** | **LpxL1 variant** | **ST** | **Clonal Complex** |
| --- | --- | --- | --- | --- | --- | --- | --- |
| 2022526 | 1 month | Meningitis w/o Shock |  |  | XI | 461 | 461 |
| 2030161 | 7 months | Meningitis w/o Shock |  |  | III | 571 | 41/44 |
| 2040084 | 1 | Shock w/o Meningitis |  |  | V | 5264 | 41/44 |
| 2050093 | 1 | Meningitis w/o Shock |  |  | V | 461 | 461 |
| 2041608 | 2 | Shock with Meningitis |  |  | Deletion* | 5413 | 41/44 |
| 2020348 | 3 | Shock with Meningitis |  |  | IV | 11 | 11 |
| 2012429 | 4 | Shock with Meningitis |  |  | III | 34 | 32 |
| 2020147 | 4 | No Shock or Meningitis |  |  | VIII | 11 | 11 |
| 2021018 | 4 | Shock with Meningitis |  |  | V | 11 | 11 |
| 2020982 | 6 | Meningitis w/o Shock | Fragile X syndrome, mental retardation |  | V | 3732 | 11 |
| 2050247 | 9 | Meningitis w/o Shock |  |  | V | 41 | 41/44 |
| 2020382 | 14 | No Shock or Meningitis |  |  | V | 41 | 41/44 |
| 2032251 | 14 | Shock w/o Meningitis |  |  | III | 7217 | 213 |
| 2031814 | 17 | No Shock or Meningitis |  |  | XII | 7056 | 41/44 |
| 2021270 | 19 | Meningitis w/o Shock |  |  | III | 461 | 461 |
| 2011799 | 21 | Meningitis w/o Shock | Osteogenesis Imperfecta |  | IV | 11 | 11 |
| 2011852 | 30 | No Shock or Meningitis | Juvenile angiofibroma nasofarynx |  | V | 11 | 11 |
| 2021197 | 39 | Shock w/o Meningitis | HIV positive |  | III | 11 | 11 |
| 2030960 | 40 |  |  |  | III | 32 | 32 |
| 2040655 | 46 | No Shock or Meningitis |  |  | Conserved Amino Acid | 571 | 41/44 |
| 2020799 | 49 | Meningitis w/o Shock |  |  | Conserved Amino Acid | 35 | 35 |
| 2040673 | 49 | Meningitis w/o Shock | Multiple Sclerose and aphasia | X | III | 41 | 41/44 |
| 2012536 | 51 | No Shock or Meningitis |  |  | III | 11 | 11 |
| 2022289 | 53 | No Shock or Meningitis | M. Crohn, Barret oesofagitis |  | X | 11 | 11 |
| 2050499 | 58 | No Shock or Meningitis | Alcohol abuses, liver failure | X | Deletion* | 5413 | 41/44 |
| 2050392 | 62 | Meningitis w/o Shock | Intracerebral haematoma |  | VII | 213 | 213 |
| 2050697 | 63 | Meningitis w/o Shock | COPD, Epilepsy, Liver disease |  | V | 41 | 41/44 |
| 2021152 | 76 | No Shock or Meningitis | COPD, AAA, meningococcal disease after 77 days of hospitalization with complicated aorta-valve replacement and 30 days on ICU | X | V | 11 | 11 |
| 2021179 | 80 | No Shock or Meningitis | Lungemboly and lunginfarction, COPD |  | IX | 3755 | 41/44 |

* Deletion in *lpxL1*-gene to large to specify
